# A172 THE LACK OF CHROMOGRANIN A PROTECTS THE COLONIC EPITHELIAL BARRIER FUNCTIONS FROM COLITIS IN MALE MICE AND EXACERBATES COLITIS IN FEMALE MICE

**DOI:** 10.1093/jcag/gwac036.172

**Published:** 2023-03-07

**Authors:** D M Tshikudi, A Diarra, J -E Ghia

**Affiliations:** 1 Immunology; 2 Pharmacology and Therapeutics , University of Manitoba; 3 IBD Clinical & Research Centre; 4 Gastrointestinal Basic Biology Research, Winnipeg, Canada

## Abstract

**Background:**

Ulcerative colitis (UC) is associated with compromised mucosal barrier function and colonic epithelial repair in a sex-dependent manner. Chromogranin A (CHGA), a pro-hormone, correlates positively with UC disease severity. In male mice, deletion of CHGA has been shown to decrease the inflammatory process; however, the effect of CHGA on mucosal barrier function and colonic epithelial repair between males and females is unknown.

**Purpose:**

We investigated whether the lack of CHGA modulates gut barrier function, mucosa integrity, and colonic epithelial repair between males and females in a mice model of colitis.

**Method:**

Male and female wild-type (WT) and CHGA (*CHGA*^*-/*-^) deficient mice (13-17 weeks old) were given 5% dextran sulfate sodium (DSS) to induce colitis or water for 5-days (n=5-8 mice per group). The disease activity index (DAI) was assessed. Colons were collected, and tumor necrosis factor (TNF)-α and IL-25 concentrations were measured by ELISA. Expression of structural and functional markers specific to epithelial cells, namely, colonocytes (Na-K-Cl cotransporter [Nkcc]1), goblet cells function (resistin-like molecule [Relm]β), and mucin [MUC]2) and stem cells (reserve Hopx, fast-cycling Lgr5, and fetal-like Ly6a cells) were evaluated by qRT-PCR.

**Result(s):**

Colitic male *CHGA*^*-/*-^ did not show significant changes in DAI compared to WT mice. Conversely, female *CHGA*^*-/*-^ mice demonstrated a trend toward higher susceptibility to colitis compared to female WT mice with increased weight loss and bleeding. This was associated with elevated levels of colonic TNF-α and IL-25 (p<0.05) in *CHGA*^*-/*-^ females compared to *CHGA*^*-/*-^ males. TNF-α levels were not different between female groups at baseline and during colitis. While colitic *CHGA*^*-/*-^ female had elevated Relmβ expression (p<0.01) compared to WT mice. No significative change was noted in Relmβ expression between female WT mice at baseline and during colitis. Similarly, Nkcc1 and Muc2 expression was not different between female groups. By contrast, male *CHGA*^*-/*-^ were less susceptible to colitis than male WT mice with elevated Nkcc1and a lower Relmβ and Muc2 expression (p<0.01). In colitis, expression of stem cell markers, Hopx and Lgr5, was markedly reduced in all groups, while male WT, *CHGA*^*-/*-^, and female WT had elevated Ly6a expression. However, the magnitude of Hopx and Ly6a expression was associated with sex. Thus, colitic male *CHGA*^*-/*-^ mice had a higher Hopx expression than male WT and female CHGA^-/ -^ mice, with a lower reduction of 1.9 compared to 4.9 and 6.6, respectively (p<0.05, 0.01, and 0.0001). While colitic male *CHGA*^*-/*-^ mice had elevated Ly6a expression (p<0.05) in contrast to female *CHGA*^*-/*-^ mice (p=0.5). The magnitude of the decrease in Lgr5 expression was not different between all groups.

**Conclusion(s):**

In the absence of CHGA, male mice preserved their colonic mucosa integrity and repair potential, while female mice suffered significant loss of mucosa integrity and repair potential during colitis.

**Disclosure of Interest:**

None Declared

